# Prevalence of Smith–Lemli–Opitz Syndrome Carriers and the Spectrum of *DHCR7* Pathogenic Variants in Representative Czech and Hungarian Population Cohorts

**DOI:** 10.3390/genes17020164

**Published:** 2026-01-30

**Authors:** Eszter Kovács, Zsuzsanna Szűcs, Miroslav Horňák, David Kubíček, Kateřina Weisová, Kateřina Veselá, Lenka Krůzová, Jan Geryk, Jan Diblík, Martina Bittóová, Milan Macek, István Balogh, Katalin Koczok

**Affiliations:** 1 Department of Medical Genetics, Faculty of Medicine, University of Debrecen, 4032 Debrecen, Hungary; kovacs.eszter@med.unideb.hu (E.K.); szucs.zsuzsanna@med.unideb.hu (Z.S.);; 2 Doctoral School of Molecular Cell and Immune Biology, Faculty of Medicine, University of Debrecen, 4032 Debrecen, Hungary; 3 Repromeda, Assisted Reproduction Centre, 625 00 Brno, Czech Republic; 4 Department of Histology and Embryology, Faculty of Medicine, Masaryk University, 625 00 Brno, Czech Republic; 5 Department of Experimental Biology, Faculty of Science, Masaryk University, 625 00 Brno, Czech Republic; 6 Institute of Applied Biotechnologies a.s., 199 00 Prague, Czech Republic; 7 Department of Biology and Medical Genetics, Second Faculty of Medicine, Charles University and Motol and Homolka University Hospital, 150 06 Prague, Czech Republic; 8 GENNET, Centre of Medical Genetics and Reproductive Medicine, 170 00 Prague, Czech Republic

**Keywords:** Smith–Lemli–Opitz syndrome, SLOS, *DHCR7*, carrier frequency

## Abstract

Background: Smith–Lemli–Opitz syndrome (SLOS) is an inborn error of cholesterol biosynthesis, caused by biallelic mutations in the *DHCR7* gene. Genotype–phenotype correlations regarding *DHCR7* variants could explain the variation in severity, ranging from in utero demise or severe SLOS to a mild phenotype. Clinical recognition can be challenging. This study aimed to determine the frequency of SLOS carriers in the Central European population, as well as the mutational spectrum of *DHCR7* in these carriers. Methods: A retrospective analysis of *DHCR7* variants was conducted using next-generation sequencing data from 55,289 individuals in Czech and Hungarian genetic laboratories. Results: The SLOS carrier frequency and the mutational spectrum of the *DHCR7* gene in its carriers were established in the Czech and Hungarian sub-cohorts. In the combined dataset, we identified causative *DHCR7* variants on 1567 alleles among 55,289 tested individuals, contributing to an SLOS carrier frequency of 2.83%. Of the 31 *DHCR7* variants detected, the c.452G>A variant was the most prevalent, accounting for 1.8% of all detected alleles in our cohorts. In contrast, the c.964-1G>C variant was more frequent in non-Finnish Europeans, as indicated by the gnomAD 4.1.0 database. The *DHCR7* mutational spectra of patients and carriers were comparable in terms of the most common variants. Conclusions: The high SLOS carrier frequency (2.83%) underscores the importance of SLOS carrier screening in Central European populations. The prevalent *DHCR7* null mutations and their potential combinations may explain the lower-than-expected prevalence of SLOS, whilst Central and Eastern European populations remain likely underrepresented in the current gnomAD database.

## 1. Introduction

Smith–Lemli–Opitz syndrome (SLOS; MIM: 270400) is a congenital disorder, inherited in an autosomal recessive manner. The severe form of this currently incurable disease can present with prenatal and postnatal growth restriction, microcephaly, moderate-to-severe intellectual disability, and multiple major and minor malformations. However, milder cases have also been described, some affected individuals may exhibit normal development and only minor malformations. It results from an inborn error of cholesterol biosynthesis caused by a deficiency of 7-dehydrocholesterol reductase (DHCR7) [1,2,3]. In addition to cholesterol deficiency, 7-dehydrocholesterol (7-DHC)-derived oxysterols are likely to contribute to disease pathophysiology [4,5].

Until recently, 182 disease-causing variants (later referred to as mutations; according to the Human Gene Mutation Database (HGMD) Professional 2025.3) have been reported in the *DHCR7* gene, which is located on chromosome 11q13.4. Although many missense mutations have been published, the two most common alleles are a splice site (c.964-1G>C) and a nonsense mutation (c.452G>A) [6].

The distribution of the common pathogenic *DHCR7* variants across Europe is uneven, showing an East–West gradient (i.e., more frequent in Eastern Europe than in the Western European dataset) for c.452G>A, in contrast with the frequency gradient of the c.964-1G>C variant, which is distributed in the opposite direction in European populations. The observed pattern of different *DHCR7* mutations in Europe provides evidence for their different origins [7]. The combined effects of founder effects, recurrent mutations, and genetic drift in shaping the distribution of *DHCR7* variants in Europe have been suggested [8].

The mutational spectrum of Czech and Hungarian SLOS patients has been previously published. Kozák et al. [9] identified the c.452G>A *DHCR7* variant as the most common, followed by c.976G>T in five unrelated SLOS families from the Czech population. Additionally, Blahakova et al. [10] conducted a four-year study involving pregnant women from the Czech Republic who were offered second-trimester biochemical screening. Based on low levels of unconjugated serum estriol, the risk of the fetus having SLOS was considered high in 456 pregnancies. In these high-risk cases, molecular genetic analysis of fetal or parental DNA samples identified six different causative *DHCR7* mutations, leading to a prenatal diagnosis of SLOS in five cases. Unfortunately, two pregnancies turned out to be biochemically false negatives, yet SLOS patients were still born. This second dataset showed, similarly to Kozák et al. [9], that the c.452G>A variant was the most frequent, with the c.964-1G>C mutation being the second most common. A study of 13 Hungarian SLOS patients also found that the c.452G>A mutation was the most prevalent, followed by the c.964-1G>C variant as the second most common allele [11].

A Canadian study examining the carrier rate and ethnic distribution of SLOS found a carrier frequency of 1.09% for the c.964-1G>C variant in *DHCR7* among Caucasian Ontarians of European descent. The authors suggested that, assuming this mutation accounts for one-third of all SLOS alleles, the carrier frequency of SLOS can be presumed to be approximately 1 in 30 for European Caucasians (95% confidence interval = 1:21 to 1:58). The estimated incidence of SLOS ranged from 1 in 1700 to 1 in 13,400 [12].

In the Polish population, the overall *DHCR7* carrier frequency was estimated to range from 1 in 24 to 1 in 31 (95% confidence interval) based on screening for the common c.452G>A and c.976G>T mutations. The incidence of SLOS is estimated to fall within the range of 1 in 2300 to 1 in 3937 [13]. A national surveillance program in Poland, conducted from 2006 to 2008, aimed to identify potential SLOS patients before and after birth. This resulted in an estimated average incidence of 1 in 83,168 live births; the average carrier frequency was calculated to be between 1 in 123 and 1 in 165 [14]. These targeted screening results also indicate that previously estimated SLOS incidence data (1 in 20,000 to 1 in 40,000 births [15]) are significantly lower than expected based solely on the carrier frequency. However, estimated carrier rates from targeted population screening for the most common *DHCR7* mutations may not accurately reflect the actual carrier frequency [16]. Therefore, comprehensive genomic testing of the *DHCR7* gene is considered a potential method to address this issue.

Cross et al. [17] reported a carrier frequency of 1.01% for pathogenic *DHCR7* mutations in four exome data sets, which included a total of 17,836 alleles. They estimated an SLOS incidence of 1 in 39,215 conceptions; however, they did not provide data on the ethnicity of the individuals. An expanded carrier screening study, which analyzed the *DHCR7* gene among others, in 23,453 individuals from diverse ethnic backgrounds, reported an overall SLOS carrier frequency of 1 in 68.2 [18]. Lazarin et al. [19] also examined results from expanded carrier screening panels in 262,399 individuals, with 210,857 screened for 13 *DHCR7* mutations via targeted genotyping, and 51,542 undergoing comprehensive mutation analysis. In this dataset, the cumulative carrier frequency was 1.87% among Northern Europeans and 1.51% among Southern Europeans.

Even though the carrier frequency of SLOS is as high as 2.3% in the well-studied Ashkenazi Jewish population, among whom the c.964-1G>C variant is the most prevalent, the disease is rarely diagnosed [20].

To our knowledge, no large-scale assessment of SLOS carrier frequency in individuals of Central European descent has been performed. Therefore, we conducted a retrospective analysis of genomic data from individuals who underwent genetic testing at laboratories in the Czech Republic and Hungary. The study aimed to determine the carrier frequency and mutational spectrum of the *DHCR7* gene in this region.

## 2. Patients and Methods

### 2.1. Patients and Data Collection

In total, data on *DHCR7* gene variants were gathered from 55,289 individuals. A retrospective analysis was performed on an anonymized combined dataset from five contributing partners.

Our SLOS carrier cohort consisted of three main groups of individuals. One group was tested for monogenic diseases other than SLOS. The *DHCR7* gene was analyzed using various methods, including a diagnostic gene panel test, clinical exome sequencing, whole-exome sequencing, or whole-genome sequencing. Genetic testing was conducted at the University of Debrecen in Hungary and at the Department of Biology and Medical Genetics at Charles University, the University Hospital Motol in the Czech Republic.

Another group of individuals requested expanded carrier screening tests to aid in their reproductive decision-making. These carrier screening tests were primarily offered by infertility clinics, therefore many of the individuals undergoing testing (or their partners) had reproductive failure. Expanded carrier screening tests were carried out at the Repromeda and GENNET laboratories, both of which are located in the Czech Republic.

Additionally, *DHCR7* variant data from 750 healthy individuals involved in the ENIGMA (Etalon of National Interpreted Genome Map of the Czech Republic) were included in the cohort. Data analysis of ENIGMA was performed at the Institute of Applied Biotechnologies and the Institute of Molecular and Translational Medicine in the Czech Republic.

Patients with a known or suspected SLOS phenotype were excluded from the analysis.

Genetic testing was performed according to the protocols of the participating laboratories.

All tested individuals have consented to genetic testing and the anonymous use of their data for research purposes, in accordance with national laws and regulations.

### 2.2. Next-Generation Sequencing

Next-generation sequencing was performed on Illumina (https://www.illumina.com/ (accessed on 17 December 2025)), BGI (https://en.genomics.cn/en-sequencplatform.html (accessed on 17 December 2025)), and MGI (https://global-mgitech.com/ (accessed on 17 December 2025)) platforms following protocols provided by the contributing laboratories. Data were aligned to the hg38 version of the human reference genome, using the canonical transcript (NM_001360.3) of the *DHCR7* gene for variant annotation. Detailed methodology is available upon request.

### 2.3. Variant Classification

*DHCR7* variants were classified according to the recommendations of the American College of Medical Genetics and Genomics (ACMG)/Association for Molecular Pathology (AMP) and its amendments [21]. Subsequent *DHCR7* carrier frequency calculations in this cohort were based on disease-causing (i.e., pathogenic or likely pathogenic) variants.

### 2.4. Estimation of SLOS Carrier Frequency Based on gnomAD 4.1.0

The disease-causing *DHCR7* gene variants identified in our cohort were entered into GeniE, the Genetic Prevalence Estimator [22], a free online tool that uses gnomAD allele frequencies to estimate the genetic prevalence of autosomal recessive diseases. For the SLOS carrier frequency calculations using the gnomAD 4.1.0 dataset, the transcript ENST00000355527.8 of the *DHCR7* gene (ENSG00000172893.18) was used, with the reference genome being its GRCh38 version.

## 3. Results

### 3.1. SLOS Carrier Frequency and DHCR7 Mutational Spectrum in SLOS Carriers

#### 3.1.1. SLOS Carrier Frequency and *DHCR7* Variant Distribution Data from the Czech Laboratories

The allele frequency data for each disease-causing *DHCR7* variant detected in the Czech population by laboratories are shown in Table 1. The combined sequencing data from four centers were obtained from a total of 53,721 individuals. Causative *DHCR7* gene variants were found on 1530 alleles. This sub-cohort included 28 different *DHCR7* variants; of these, 24 were classified as pathogenic, and four as likely pathogenic. The most common mutation identified was c.452G>A, accounting for 65% of cases. The second most frequent variant was c.964-1G>C, making up 24% of the *DHCR7* variants in the Czech dataset. Overall, among the 53,721 individuals tested in the Czech Republic, 2.85% were found to be carriers of SLOS.

#### 3.1.2. Hungarian SLOS Carrier Frequency and *DHCR7* Variant Distribution

The Hungarian SLOS carrier dataset is shown in Table 2.

Among the 1568 Hungarian individuals tested in our cohort, we identified a causative *DHCR7* gene variant in 37 cases. Seven of the variants identified (present on 36 alleles) were classified as pathogenic, and one was classified as likely pathogenic. The c.452G>A variant was the most prevalent in the Hungarian sub-cohort (62%). 2.36% of tested Hungarian individuals were found to be SLOS carriers.

#### 3.1.3. Combined SLOS Carrier Frequency and *DHCR7* Variant Dataset

Overall, we identified 31 unique causative (pathogenic or likely pathogenic) *DHCR7* variants, found on 1567 alleles across 55,289 individuals tested. The most common variant was c.452G>A, present in 1017 individuals and representing 65% of SLOS carriers in this group. The second most frequent variant was c.964-1G>C, found on 363 alleles, accounting for 23% of SLOS carriers in the combined cohort. Analysis of the combined Czech and Hungarian population data showed that 2.83% of the individuals tested were carriers of SLOS. Additionally, we compared the SLOS carrier frequency of *DHCR7* variants in our cohort with data from the gnomAD non-Finnish European ancestry group.

Table 3 presents the combined *DHCR7* variant and allele frequency data in detail, alongside the gnomAD 4.1.0 allele frequencies of the detected variants worldwide and in the non-Finnish European population.

### 3.2. Estimation of SLOS Carrier Frequency Based on gnomAD 4.1.0 and Comparison with Carrier Frequency Detected in the Cohort

Thirty of 31 *DHCR7* variants detected in our carrier cohort were entered into GeniE, the Genetic Prevalence Estimator tool, to calculate the estimated SLOS carrier frequency in the non-Finnish European ancestry group, based on gnomAD 4.1.0 allele frequency data. All variants but c.1066_1067del were present in the gnomAD 4.1.0 database; therefore, this variant was not taken into account when calculating the cumulative carrier frequency.

GeniE calculated the cumulative SLOS carrier frequency based on gnomAD 4.1.0 data to be 1:45 in the non-Finnish European population, which equates to an SLOS carrier frequency of approximately 2%, compared to the nearly 3% observed in our carrier cohort.

### 3.3. Comparing Mutational Spectra in SLOS Carriers and SLOS Patients

We compared the allele frequencies of the *DHCR7* variants detected in Czech and Hungarian SLOS patients with those in the carrier cohort. *DHCR7* variant frequencies in the previously published Czech and Hungarian SLOS patients [9,10,11] are summarized in Table 4 and Table 5, respectively.

## 4. Discussion

Analysis of *DHCR7* gene variants in a large combined dataset of 55,289 Czech and Hungarian individuals identified 1567 disease-causing alleles, corresponding to a carrier frequency of 2.83% for SLOS.

Our combined cohort is representative of Central European populations. Compared to the SLOS carrier frequencies observed in Northern Europeans (1.87%) and Southern Europeans (1.51%) [19], the 2.83% SLOS carrier frequency found in our Central European cohort stands out.

The two most common *DHCR7* variants, the c.452G>A nonsense mutation and the c.964-1G>C splice site variant, were found in 65% and 23% of SLOS carriers, respectively, across the entire cohort. The high rate of null mutations and their possible combinations may explain the lower-than-expected prevalence of SLOS, likely due to early in utero death, as previous clinical studies on genotype–phenotype correlations involving *DHCR7* variants suggest [23,24,25,26]. Besides the suspected high intrauterine mortality rate of affected embryos or fetuses in the first trimester, the difference between the predicted and observed SLOS incidence might be caused by: (i) SLOS being clinically underdiagnosed, (ii) reduced fertility among SLOS carriers, and/or iii) incomplete penetrance of hypomorphic pathogenic variants [14,16].

As the combination of the common c.976G>T and c.452G>A variants has been hypothesized to result in miscarriage, a Polish study examined the possible connection between *DHCR7* variant carrier status and recurrent miscarriage. The research involved 380 women who had experienced at least two miscarriages and 100 control individuals. The c.976G>T variant was not found in the cohort, but the c.452G>A variant was present in 5.8% in women with miscarriage, compared to the 2% allele frequency in the control group. However, statistical analysis revealed that the difference in prevalence of the c.452G>A variant between the recurrent miscarriage and control groups was not significant [27]. To date, no large-scale studies have been conducted to further evaluate the connection between recurrent miscarriage and SLOS carrier status. Therefore, it would be worth performing studies with a larger number of cases to address this potential clinical implication.

A comparison of data on *DHCR7* variant frequencies in our cohort and gnomAD revealed notable differences. The most common c.452G>A mutation was found with an allele frequency of 1.8% in our cohort. In contrast, it was present in only 0.07% of alleles in the gnomAD aggregated database and 0.09% in the gnomAD European non-Finnish population. The second most frequent variant in our cohort, c.964-1G>C, was the most common in the gnomAD non-Finnish population, found in 0.9% of alleles. Conversely, its allele frequency was 0.7% in both the gnomAD aggregated database and our cohort. The differences in allele frequencies are because Eastern and Central European countries did not contribute samples to the gnomAD dataset.

We compared the mutational spectrum of disease-causing *DHCR7* variants in SLOS carriers with the previously published mutational spectrum of Czech [9,10] and Hungarian [11] SLOS patients. We found that the c.452G>A variant was present on most alleles in our recent SLOS carrier cohort, as well as in the Czech and Hungarian SLOS cases previously described. The second and third most common *DHCR7* variants (c.964–1G>C and c.976G>T, respectively) in SLOS carriers also matched those reported in patient cohorts. Some disease-causing *DHCR7* variants identified in SLOS patients were not found in our combined carrier cohort. Specifically, the c.1139G>A, c.861C>A/G, and c.546G>T/C variants identified in Czech SLOS patients, along with the c.1328G>A, c.730G>A, and c.374A>G mutations previously seen in Hungarian SLOS patients, were missing from our carrier dataset. One possible explanation is the presence of unique *DHCR7* variants or the fact that only a small number of SLOS patients have been documented in the literature; therefore, the data published thus far may not fully capture the complete mutational spectrum.

Based on scientific research, there is also evidence that being a carrier of a pathogenic *DHCR7* gene mutation may potentially have additional medical implications. Some medications, such as aripiprazole, that interfere with sterol biosynthesis can also increase 7-DHC levels. If a pregnant SLOS carrier takes such medications, they could potentially harm the developing fetus [28,29,30].

Considering the high carrier frequency and possible clinical implications, it is understandable that *DHCR7* was included in the tier 3 list of genes recommended for carrier screening by the American College of Medical Genetics and Genomics [31].

We acknowledge the study’s limitations. First, various methods were used for *DHCR7* mutational testing in the studied cohorts. Most of these tests, such as diagnostic gene panels, clinical exome sequencing, whole-exome sequencing, and whole-genome sequencing are based on next-generation sequencing. They can detect variants in all coding exons of *DHCR7,* as well as in flanking intronic regions. However, some versions of extended carrier screening tests only included probes for the well-known *DHCR7* pathogenic mutations, potentially missing some causative variants.

We also admit the limited capability of next-generation sequencing methods for detecting copy number variations (CNVs). As approximately 4% of *DHCR7* mutations can be detected using gene-targeted deletion/duplication analysis [2], these pathogenic CNVs [32] may have been missed by our approach.

Information about the ethnicity of each tested individual in the collaborating Czech laboratories was not available, but most of the individuals tested were Czech nationals.

## 5. Conclusions

The notable 2.83% SLOS carrier frequency found in our Central European cohort highlights the need for SLOS carrier screening in Czech, Hungarian, and broader Central European populations, in line with the ACMG’s recommendation [29].

The prevalent *DHCR7* null mutations and their potential combinations may explain the lower-than-expected prevalence of SLOS in Central Europe.

It would be worthwhile to perform larger studies to investigate the potential connection between recurrent miscarriage and SLOS carrier status.

Central and Eastern European populations are likely underrepresented in the current gnomAD database.

## Figures and Tables

**Table 1:** Genotype of the SLOS carriers and *DHCR7* allele frequencies in Czechia.

Genotype *	Effect on Protein	ACMG/AMP Classification	Allele Count	Allele Frequency in the Sub-Cohort
c.452G>A	p.(Trp151Ter)	P	994/53,721	0.018503006
c.964-1G>C	splicing affected	P	361/53,721	0.006719905
c.976G>T	p.(Val326Leu)	P	82/53,721	0.001526405
c.326T>C	p.(Leu109Pro)	P	15/53,721	0.00027922
c.1337G>A	p.(Arg446Gln)	P	13/53,721	0.000241991
c.841G>A	p.(Val281Met)	P	10/53,721	0.000186147
c.724C>T	p.(Arg242Cys)	P	10/53,721	0.000186147
c.964-1G>T	splicing affected	P	6/53,721	0.000111688
c.355del	p.(His119IlefsTer8)	P	6/53,721	0.000111688
c.89G>C	p.(Gly30Ala)	P	5/53,721	9.30735 × 10^−5^
c.725G>A	p.(Arg242His)	P	4/53,721	7.44588 × 10^−5^
c.470T>C	p.(Leu157Pro)	P	3/53,721	5.58441 × 10^−5^
c.1066del	p.(His356ThrfsTer57)	P	2/53,721	3.72294 × 10^−5^
c.506C>T	p.(Ser169Leu)	P	2/53,721	3.72294 × 10^−5^
c.1342G>A	p.(Glu448Lys)	P	1/53,721	1.86147 × 10^−5^
c.1295A>G	p.(Tyr432Cys)	P	1/53,721	1.86147 × 10^−5^
c.1228G>A	p.(Gly410Ser)	P	1/53,721	1.86147 × 10^−5^
c.1219A>T	p.(Asn407Tyr)	P	1/53,721	1.86147 × 10^−5^
c.1055G>A	p.(Arg352Gln)	P	1/53,721	1.86147 × 10^−5^
c.1054C>T	p.(Arg352Trp)	P	1/53,721	1.86147 × 10^−5^
c.385_412+5del	splicing affected	P	1/53,721	1.86147 × 10^−5^
c.151C>T	p.(Pro51Ser)	P	1/53,721	1.86147 × 10^−5^
c.3G>A	p.(Met1?)	P	1/53,721	1.86147 × 10^−5^
c.1A>G	p.(Met1?)	P	1/53,721	1.86147 × 10^−5^
c.627-1G>A	splicing affected	LP	3/53,721	5.58441 × 10^−5^
c.1066_1067del	p.(His356ProfsTer200)	LP	2/53,721	3.72294 × 10^−5^
c.1211G>A	p.(Arg404His)	LP	1/53,721	1.86147 × 10^−5^
c.739G>A	p.(Ala247Thr)	LP	1/53,721	1.86147 × 10^−5^

* All variants were present in heterozygous form; P—pathogenic, LP—likely pathogenic.

**Table 2:** Genotype of the SLOS carriers and *DHCR7* allele frequencies in Hungary.

Genotype *	Effect on Protein	ACMG/AMP Classification	Allele Count	Allele Frequency in the Sub-Cohort
c.452G>A	p.(Trp151Ter)	P	23/1568	0.014668367
c.470T>C	p.(Leu157Pro)	P	4/1568	0.00255102
c.89G>C	p.(Gly30Ala)	P	3/1568	0.001913265
c.964-1G>C	splicing affected	P	2/1568	0.00127551
c.326T>C	p.(Leu109Pro)	P	2/1568	0.00127551
c.1190C>T	p.(Ser397Leu)	P	1/1568	0.000637755
c.91C>T	p.(Arg31Cys)	P	1/1568	0.000637755
c.520T>G	p.(Phe174Val)	LP	1/1568	0.000637755

* All variants were present in heterozygous form; P—pathogenic, LP—likely pathogenic.

**Table 3:** Combined SLOS carrier frequency data from the two sub-cohorts and gnomAD 4.1.0 allele frequency of the detected variants.

Genotype *	Effect on Protein	ACMG/AMP Classification	Allele Count	Allele Frequency in the Total Cohort	gnomAD 4.1.0 Aggregated Allele Frequency	gnomAD 4.1.0 European (Non-Finnish) Allele Frequency
c.452G>A	p.(Trp151Ter)	P	1017/55,289	0.018394256	0.0007107	0.0008975
c.964-1G>C	splicing affected	P	363/55,289	0.006565501	0.007446	0.009207
c.976G>T	p.(Val326Leu)	P	82/55,289	0.001483116	0.00002620	0.00003320
c.326T>C	p.(Leu109Pro)	P	17/55,289	0.000307475	0.000001240	0.000001695
c.1337G>A	p.(Arg446Gln)	P	13/55,289	0.000235128	0.00003721	0.00004407
c.841G>A	p.(Val281Met)	P	10/55,289	0.000180868	0.00003037	0.00001017
c.724C>T	p.(Arg242Cys)	P	10/55,289	0.000180868	0.0001915	0.0002381
c.89G>C	p.(Gly30Ala)	P	8/55,289	0.000144694	0.0001227	0.0001653
c.470T>C	p.(Leu157Pro)	P	7/55,289	0.000126607	0.00002726	0.00003305
c.964-1G>T	splicing affected	P	6/55,289	0.000108521	0.00004438	0.00004599
c.355del	p.(His119IlefsTer8)	P	6/55,289	0.000108521	0.000008675	0.00001102
c.725G>A	p.(Arg242His)	P	4/55,289	7.23471 × 10^−5^	0.00004275	0.00005085
c.1066del	p.(His356ThrfsTer57)	P	2/55,289	3.61736 × 10^−5^	0.00001923	0.00002459
c.506C>T	p.(Ser169Leu)	P	2/55,289	3.61736 × 10^−5^	0.00002354	0.00002712
c.1342G>A	p.(Glu448Lys)	P	1/55,289	1.80868 × 10^−5^	0.0001166	0.0001534
c.1295A>G	p.(Tyr432Cys)	P	1/55,289	1.80868 × 10^−5^	6.204 × 10^−7^	8.477 × 10^−7^
c.1228G>A	p.(Gly410Ser)	P	1/55,289	1.80868 × 10^−5^	0.00003352	0.00002966
c.1219A>T	p.(Asn407Tyr)	P	1/55,289	1.80868 × 10^−5^	0.000008689	0.00001187
c.1190C>T	p.(Ser397Leu)	P	1/55,289	1.80868 × 10^−5^	0.00001305	0.00001356
c.1055G>A	p.(Arg352Gln)	P	1/55,289	1.80868 × 10^−5^	0.00001799	0.00001272
c.1054C>T	p.(Arg352Trp)	P	1/55,289	1.80868 × 10^−5^	0.00001799	0.00001866
c.385_412+5del	splicing affected	P	1/55,289	1.80868 × 10^−5^	0.00002796	0.00002634
c.151C>T	p.(Pro51Ser)	P	1/55,289	1.80868 × 10^−5^	0.000001243	0.000001698
c.91C>T	p.(Arg31Cys)	P	1/55,289	1.80868 × 10^−5^	0.00003346	0.00003899
c.3G>A	p.(Met1?)	P	1/55,289	1.80868 × 10^−5^	0.00001301	0.00001610
c.1A>G	p.(Met1?)	P	1/55,289	1.80868 × 10^−5^	0.00001983	0.00001356
c.627-1G>A	splicing affected	LP	3/55,289	5.42603 × 10^−5^	0.000005579	0.000006782
c.1066_1067del	p.(His356ProfsTer200)	LP	2/55,289	3.61736 × 10^−5^	not present	not present
c.1211G>A	p.(Arg404His)	LP	1/55,289	1.80868 × 10^−5^	0.000008700	0.000009326
c.739G>A	p.(Ala247Thr)	LP	1/55,289	1.80868 × 10^−5^	0.000003717	0.000001695
c.520T>G	p.(Phe174Val)	LP	1/55,289	1.80868 ×10^−5^	0.00001053	0.00001102

* All variants were present in heterozygous form; P—pathogenic, LP—likely pathogenic.

**Table 4:** *DHCR7* allele frequency data in Czech SLOS patients, based on the publications of Kozák et al. [9] and Blahakova et al. [10].

Variant	Allele Count	Allele Frequency in the Two Czech SLOS Patient Cohorts
c.452G>A	10/24	0.417
c.964–1G>C	4/24	0.167
c.976G>T	3/24	0.125
c.1139G>A	2/24	0.083
c.1337G>A	1/24	0.042
c.861C>A/G *	1/24	0.042
c.546G>T/C **	1/24	0.042
c.470T>C	1/24	0.042
c.89G>C	1/24	0.042

* The p.N287K variant was described initially only at the protein level by Blahakova et al. (2007) [10]. It can be associated with two different variants on the nucleotide level, i.e., c.861C>A and c.861C>G. Both c.861C>A and c.861C>G variants in the *DHCR7* gene can be classified as disease-causing. ** The p.W182C variant was described initially only at the protein level by Blahakova et al. (2007) [10]. It can be associated with two different variants on the nucleotide level, i.e., c.546G>T and c.546G>C. Both c.546G>T and c.546G>C variants in the *DHCR7* gene can be classified as disease-causing.

**Table 5:** *DHCR7* allele frequency of unrelated Hungarian SLOS patients, based on the work of Balogh et al. [11].

Variant	Allele Count	Allele Frequency in the Hungarian SLOS Patient Cohort
c.452G>A	6/24	0.250
c.964–1G>C	4/24	0.167
c.976G>T	3/24	0.125
c.1295A>G	2/24	0.083
c.1190C>T	2/24	0.083
c.1328G>A	1/24	0.042
c.740C>T	1/24	0.042
c.730G>A	1/24	0.042
c.725G>A	1/24	0.042
c.374A>G	1/24	0.042
c.326T>C	1/24	0.042

## Data Availability

The raw data supporting the conclusions of this article will be made available by the authors on request.
